# 临床疑诊肺癌纵隔淋巴结转移EBUS-TBNA阴性患者的处理

**DOI:** 10.3779/j.issn.1009-3419.2019.04.04

**Published:** 2019-04-20

**Authors:** 永健 留, 闽江 陈, 雪峰 孙, 池 邵, 燕 徐, 勇 陈, 媛媛 赵, 静 赵, 孟昭 王

**Affiliations:** 100730 北京，中国医学科学院，北京协和医学院，北京协和医院呼吸与危重症医学科 Department of Respiratory and Critical Care Medicine, Peking Union Medical College Hospital, Chinese Academy of Medical Science & Peking Union Medical College, Beijing 100730, China

**Keywords:** 超声内镜引导下的经支气管针吸活检, 病理阴性, 肺肿瘤, Endobronchial ultrasound guided transbronchial needle aspiration, Non-specific pathology, Lung neoplasms

## Abstract

**背景与目的:**

超声气管镜针吸活检（endobronchial ultrasound guided tranbronchial needle aspiration, EBUS-TBNA）是肺癌诊断和分期的重要手段，但经活检阴性结果的患者后续处理尚无标准流程。本文通过分析来自单中心的临床疑诊肺癌纵隔淋巴结转移但EBUS-TBNA病理结果阴性患者，以探讨此类患者处理方式。

**方法:**

对北京协和医院2010年9月-2016年12月进行EBUS-TBNA的1, 412例患者资料进行分析，选取临床疑诊肺癌纵隔淋巴结转移但EBUS-TBNA病理诊断阴性的患者51例进行回顾性分析。

**结果:**

入选51例患者按临床情况和后续处理方式分为以下5组：①经同一次气管镜下其他检查组（9例）：该组患者大多（8例）存在镜下异常表现，通过活检、毛刷、灌洗或经支气管镜肺活检（transbronchial lung biopsy, TBLB）取得明确诊断；②再次EBUS-TBNA组（11例）：该组患者气管粘膜及管腔大致正常，再次行EBUS-TBNA取得诊断；③手术治疗组（6例）：该组患者因EBUS结果除外纵隔淋巴结转移，接受手术治疗。其中5例术后确诊无淋巴结转移癌；④进行其他病理检查组（15例）：该组患者有其他部位转移，针对可能的转移灶进行计算机断层扫描（computed tomography, CT）引导下穿刺、淋巴结活检等确诊。⑤随访组（10例）：该组患者未进行其他有创检查，中位随访时间38个月，其中1例随访中诊断为淋巴瘤。

**结论:**

对于经EBUS-TBNA未能确定诊断而临床怀疑肺癌的患者，应该根据患者的具体情况，综合多种方式进行诊断。对于暂时无法确诊的患者，仍需要长期随访。

## Abstract (en)

**Background and objective:**

Endobronchial ultrasound guided transbronchial needle aspiration (EBUS-TBNA) is well known as an important technique for diagnosis and staging of lung cancer. But a standard protocol to deal with patients who have a negative pathology result still needs to be defined. Herein, we describe the subsequent procedures of these patients in a single center.

**Methods:**

A total of 1, 412 patients with clinical suspected lung cancer and mediastinal metastasis who underwent EBUS-TBNA were collected between September 2010 and December 2016. Among them, 51 patients with nonspecific pathology result were included and retrospectively analyzed.

**Results:**

The 51 patients were stratified into five groups by clinical characterize and follow-up procedures: (1) Diagnosed by other bronchoscopy procedures group (9 cases). Abnormalities of tracheobronchial tree were found during visual examination in the majority of patients (8 cases). Biopsy, endobronchial brushing, bronchoalveolar lavage, and transbronchial lung biopsy (TBLB) were used to get a specific diagnosis. (2) EBUS-TBNA re-biopsy group (11 cases). Patients in this group had normal mucosal appearance and airway lumen. Re-biopsy were performed on patients in this group. (3) Surgery group (6 cases). Patients underwent surgery after negative result of EBUS-TBNA. Five of them were confirmed with non-nodal metastasis after surgery. (4) Underwent other pathology diagnosis group (15 cases). patients in this group had other metastasis sites besides midiastinal lymph node. Computed tomography (CT)-guided fine-needle aspiration and lymph node biopsy were performed. (5) Follow-up group (10 cases). None invasive procedure was used in this group. The median follow up time was 38 months. One patient was diagnosed lymphoma during the follow up.

**Conclusion:**

Diagnostic procedures should be chosen based on the clinical character in EBUS-TBNA negative patients with suspected lung cancer. Long time follow-up is very important in patients whose diagnosis is apparently unknown.

随着支气管镜下超声影像的发展，该技术已经成为呼吸病学中一项重要的微创技术。目前超声支气管镜（endobronchial ultrasound, EBUS）可分为凸面超声和小探头超声两种，分别于1990年和2004年进入临床使用^[1, 2]^。其中经超声气管镜引导下针吸活检（endobronchial ultrasound guided transbronchial needle aspiration, EBUS-TBNA）结合了超声及细针穿刺活检，是对于纵隔肿大淋巴结进行病理诊断一项重要手段。该技术对肺癌纵隔淋巴结转移的诊断敏感性、特异性和准确性均超过了90%，同时与纵隔镜相比操作简便和创伤更小，易于被患者接受。近年来，多个研究提示EBUS-TBNA在肺癌诊断和淋巴结分期、结节病以及结核等疾病的诊断中有重要作用^[3-5]^，对肺癌纵隔淋巴结转移评估也写进了多个肺癌临床诊治指南。但是对于拟诊肺癌纵隔淋巴结转移的患者EBUS-TBNA病理诊断阴性时应该如何处理呢?本文进行了相关的探讨。

## 1 资料与方法

### 1.1 研究人群

回顾性分析北京协和医院自2010年9月-2016年12月接受EBUS-TBNA检查的患者共1, 412例。最终诊断肺部恶性疾病747例（53%），结节病353例（25%），其他诊断包括反应性增生淋巴结、结核等感染、囊肿、胸腔内甲状腺等。EBUS-TBNA诊断的总体准确性为95%，诊断肺癌的敏感性为95%，特异性为100%。本研究入组的患者为拟诊肺癌纵隔淋巴结转移的患者但EBUS-TBNA病理诊断阴性，入组条件为：①计算机断层扫描（computed tomography, CT）提示肺内病灶同时合并纵隔淋巴结肿大；②正电子发射计算机断层显像（positron emission tomography/CT, PET/CT）提示肺恶性肿瘤合并纵隔淋巴结转移；如未行PET/CT，则需要有提示“远处转移”的证据，如骨破坏、脑病灶、肝脏或肾上腺病灶等。③接受了EBUS-TBNA，但病理诊断为阴性（未找到瘤细胞）。④病例资料齐全，随访信息完整。符合上述条件的共51例患者。收集患者的性别、年龄、气管镜下表现、气管镜相关检查结果、EBUS-TBNA取材部位、穿刺针数、病理诊断、随访等资料并进行分析。本回顾性研究得到了北京协和医院伦理委员会批准。

### 1.2 操作方法和仪器设备

局麻或全麻下进行操作，首先应用日本奥林巴斯公司生产的可弯曲电子支气管镜（型号：Olympus BF-260）进行观察，EBUS-TBNA应用超声电子支气管镜（支气管镜型号OlympusUC-260-FW超声主机Olympus EU-ME1）以及一次性细胞学穿刺针（22 G-NA- 201SX-4022; Olympus）进行纵隔淋巴结穿刺。取得的组织标本经福尔马林固定24 h后石蜡包埋切片，细胞学标本涂片应用95%乙醇固定。穿刺针数取决于获得明确的组织条或患者的耐受情况决定。

### 1.3 诊断标准

患者最终的诊断确定包括：①恶性肿瘤的诊断必须是病理学诊断，标本来源包括EBUS-TBNA，也包括其他方法，如CT引导下穿刺、淋巴结活检或手术等；②结节病的诊断参照中华结核和呼吸杂志报道的结节病诊断标准；③肺结核必须有明确的抗酸染色阳性或上皮样肉芽肿伴干酪样坏死；④其他良性疾病符合相关诊断标准。

### 1.4 患者的分析

根据患者的具体情况，将患者分为五组：组1：同时进行的支气管镜下其他检查明确了诊断；组2：临床考虑肿瘤，再次进行了EBUS-TBNA；组3：考虑肿瘤同时无其他部位转移，接受了手术治疗；组4：考虑肿瘤同时有其他部位转移，进行了进一步病理检查；组5：考虑良性非特异性炎症，接受了随访。每组患者的情况进行了描述性统计。

## 2 结果

### 2.1 患者情况

入组患者的基本情况和每组患者的情况见表。可见该组人群有一定特点，小于60岁的患者为34例（66.7%），镜下正常或仅表现为粘膜充血的患者为44例（86.3%）。穿刺的常见部位为气管旁淋巴结（17例，33.3%）和隆突下淋巴结（27例，52.9%），穿刺的针数多为2针-3针（43例，84.3%）。

### 2.2 每组患者的进一步诊治情况

组1中虽然EBUS-TBNA结果阴性，但同一次支气管镜下其他的检查明确了诊断的病例共9例。镜下可见新生物或结节的患者2例，粘膜活检均明确了诊断，分别为肺鳞癌和肺未分化非小细胞肺癌。镜下表现为粘膜充血的患者6例，其中6例均进行了粘膜毛刷、5例进行了粘膜活检、5例进行了支气管肺泡灌洗、4例进行了经支气管镜肺活检（transbronchial lung biopsy, TBLB），结果粘膜活检诊断肺腺癌2例、毛刷诊断腺癌1例、支气管肺泡灌洗诊断腺癌1例、粘膜活检诊断肺结核1例、TBLB诊断肺结核1例。镜下正常患者1例，通过TBLB诊断肺结核。粘膜活检为肺癌的4例患者均进行了免疫组化检查。

组2中临床考虑肿瘤，再次进行了EBUS-TBNA检查共11例。通过重复EBUS-TBNA检查，确诊肺癌8例，均为腺癌，其中7例病理组织可以完成表皮生长因子（epithelial growth factor receptor,* EGFR*）基因突变检测，*EGFR*基因突变阳性4例。诊断肺结核2例，肺结节病1例。

组3中临床考虑肿瘤同时检查评估无其他部位转移，而是否有纵隔淋巴结转移不能确定，患者接受了手术治疗共6例。术后病理显示肺癌不伴有纵隔淋巴结转移4例，其中腺癌3例和小细胞肺癌1例；肺癌伴有纵隔淋巴结转移1例，病理为鳞癌；肺良性疾病1例，病理显示为肺慢性炎。

组4中临床考虑肿瘤同时有其他部位转移，进行了进一步病理检查共15例。其中肺癌5例，分别为经皮肺穿刺诊断肺腺癌2例、重复气管镜粘膜活检诊断肺腺癌1例、锁骨上淋巴结活检诊断肺腺癌1例和纵隔镜淋巴结活检诊断肺小细胞肺癌1例。其他恶性肿瘤5例，分别为经皮肺穿刺诊断霍奇金淋巴瘤1例、颈部淋巴结活检诊断霍奇金淋巴瘤1例、胃镜粘膜活检诊断胃癌1例、肾上腺手术诊断恶性嗜铬细胞瘤1例和肾脏病灶穿刺活检诊断炎性肌纤维母细胞瘤1例。诊断良性疾病5例，分别为颈部淋巴结活检诊断坏死性淋巴结炎2例和成人Still’s病1例、纵隔镜淋巴结活检诊断慢性炎症1例和心脏手术瓣膜活检为真菌性心瓣膜炎1例。

组5中临床考虑良性非特异性炎症，接受了随访共10例，中位随访时间38个月（18个月-75个月）。其中1例随访18个月后，出现轻度皮肤斑丘疹和低热，进一步检查发现颈部和腹膜后淋巴结肿大，通过颈部淋巴结活检诊断为霍奇金淋巴瘤。其他9例患者病情稳定。

**1:** 各组患者的一般特征 Clinical characteristics of patients in 5 groups

Index		Group 1	Group 2	Group 3	Group 4	Group 5	Total
Number		9	11	6	15	10	51
Gender							
	Male	5	6	4	8	5	28
	Female	4	5	2	7	5	23
Age(yr)							
	≥60	3	5	4	3	2	17
	< 60	6	6	2	12	8	34
Bronchoscpy menifestation							
	Normal	1	2	3	6	3	15
	Congestion	6	8	2	8	5	29
	Neoplasm	2	1	1	1	2	7
Biopsy							
	Undone	2	8	6	11	9	36
	Negative	2	3	0	4	1	10
	Positive	5	0	0	0	0	5
Brush							
	Negative	8	11	6	15	10	50
	Positive	1	0	0	0	0	1
Location of lymph node							
	Parabronchial	4	3	1	5	4	17
	N5	0	0	1	1	0	2
	N7	4	7	2	9	5	27
	N8	1	1	2	0	1	5
Times of TBNA							
	2	6	2	4	12	6	30
	3	3	3	2	3	2	13
	≥4	0	6	0	0	2	8
TBNA: transbronchial needle aspiration.							

### 2.3 EBUS-TBNA的安全性

所有患者均可耐受检查，主要不良反应为操作过程中气道刺激和术后少量咯血（痰中带血），在术后1天均自行好转。检查时间稍延长。无患者出气胸、纵隔气肿以及大咯血等严重并发症。

## 3 讨论

EBUS-TBNA作为一种微创操作，在纵隔疾病的诊断中占据越来越重要的地位。操作中通过超声探头的实时监测，可更加精确定位目标纵隔淋巴结，同时降低了穿刺损伤的可能。EBUS-TBNA的穿刺范围包括纵隔淋巴结2组、4组、7组，同时对明显增大的5组和部分8组也可以进行穿刺。与胸腔镜相比，可同时对双侧纵隔肺门淋巴结进行活检；从安全性和便捷性看，大多数EBUS-TBNA可采取门诊局麻操作的方式，与胸科手术相比更加简便微创。因此，更加易于为患者接受。EBUS-TBNA已经取代胸腔镜和纵隔镜的作用，逐渐成为一项首选的纵隔疾病检查手段，在多个指南中进行了推荐。本研究中，对拟诊肺癌纵隔淋巴结转移，但EBUS-TBNA阴性的情况进行了分析，并提出了相应的处理方案。

本文共从1, 412例EBUS-TBNA检查中分析了51例阴性患者的进一步诊治，可见总体该检查的敏感性和特异性都很高，与国外的报道^[6-8]^相似，本组患者共最终诊断肺部恶性疾病747例，通过重复EBUS-TBNA仅有1例手术证明为真正的假阴性。说明EBUS-TBNA是肺癌患者取得病理诊断的一项重要途径。本文中11例再次EBUS-TBNA均明确了诊断，包括结节病1例^[9-11]^，特别是再次EBUS-TBNA诊断肺癌的8例患者中，7例（87.5%）取得的标本可以进行EGFR基因检测，其中4例（57.1%）EGFR突变阳性，患者接受了EGFR-TKI的治疗，说明了EBUS-TBNA对诊治的重要性。

EBUS-TBNA对诊断纵隔淋巴结固然非常重要，但是其他方法也不能忽视，特别在组1中支气管镜下其他的检查明确了诊断的病例共9例。即使是粘膜水肿时的粘膜活检、毛刷、支气管肺泡灌洗、TBLB等均有助于明确诊断，包括恶性和良性疾病。因此在气管镜检查中应该多种手段并用。

对于EBUS-TBNA阴性患者，临床处理时应该综合考虑患者情况，如果通过检查，包括PET/CT、头部增强MRI、骨核素显像、胸腹部增强CT等，未发现其他病灶，如果疑诊恶性疾病，可以考虑手术治疗，本文中接受了手术治疗共6例。结果术后病理显示肺癌不伴有纵隔淋巴结转移4例，患者从手术中获益。只有1例EBUS-TBNA假阴性。如果有其他病灶，则建议进一步病理检查，本文中共15例进行了进一步病理检查。活检部位和方法根据病灶位置决定，结果显示肺癌5例、其他恶性肿瘤5例和良性疾病5例，也充分说明了进一步检查的必要性。

如果临床考虑良性非特异性炎症，特别是患者无症状而且仅有纵隔淋巴结肿大，影像学上有淋巴结钙化点或TBNA病理可见碳末沉积时，可以定期随访，本文中9例（90%）接受了长期随访，无病情变化。但有1例随访18月后诊断了霍奇金淋巴瘤，提示了随访的重要性^[12-14]^。

综上所述，对于拟诊肺癌纵隔淋巴结转移但EBUS-TBNA阴性的患者，应该根据患者的具体情况，综合多种方法进行诊断和随访。
